# Mechanical and Bond Performance of Alkali-Activated Slag Concrete Incorporating Natural and Recycled Diatoms

**DOI:** 10.3390/ma19091815

**Published:** 2026-04-29

**Authors:** Carlos Parra, Isabel Miñano Belmonte, Mariano Calabuig Soler, Francisco Benito, Carlos Rodriguez, Víctor Martinez Pacheco, José María Mateo, Elvira Carrión, Pilar Hidalgo Torrano

**Affiliations:** 1 Architecture and Building Technology Department, Universidad Politécnica de Cartagena (Member of European University of Technology), 30201 Murcia, Spain; isabel.minano@upct.es (I.M.B.); mariano.calabuig@upct.es (M.C.S.); francisco.benito@upct.es (F.B.); josem.mateo@upct.es (J.M.M.); elvira.carrion@upct.es (E.C.); 2 Department of Sustainable Construction, Centro Tecnológico de la Construcción, 30500 Molina de Segura, Spain; crodriguez@ctcon-rm.com; 3 Research and Development Department, Cementos La Cruz, 30640 Abanilla, Spain; vmartinez@cementoslacruz.com (V.M.P.); phidalgo@cementoslacruz.com (P.H.T.)

**Keywords:** alkali-activated concrete, alkali-activated slag, diatomaceous earth, recycled diatoms, bond strength, pull-out test, flexural behaviour

## Abstract

Alkali-activated concrete can reduce reliance on Portland cement by valorizing industrial by-products. This study evaluates slag-based alkali-activated concretes incorporating natural diatomaceous earth (M2, M3) and residual diatomaceous earth from industrial filtration (V6–V7), benchmarked against an OPC reference. The experimental program measures compressive, tensile and flexural strengths and elastic modulus, and examines steel–concrete bond behavior through bond stress–slip response at multiple slip levels. Member-level performance is assessed using reinforced beams tested under four-point bending, and cracking is compared in the constant-moment region using crack number and average spacing derived from post-test observations. Results show that diatom-based alkali-activated mixtures can achieve mechanical performance comparable to OPC concrete, with clear dependence on diatom source and mixture design. Bond response is markedly mixture-dependent and cannot be inferred from compressive strength alone. All beams exhibited flexural behavior suitable for structural applications, with the RV6 mixture providing the most favorable overall response among the tested members. These findings support the feasibility of residual diatomaceous earth as a viable component in structural alkali-activated concretes.

## 1. Introduction

The increasing demand for housing and infrastructure is expected to drive unprecedented construction activity in the coming decades [1,2,3,4]. Since structural concrete will remain a dominant material, reducing the environmental footprint of its binder phase is a central challenge. Ordinary Portland cement (OPC) production is associated with significant CO_2_ emissions, largely linked to clinker manufacture (calcination of limestone and high-temperature processing) [5,6,7,8]. Therefore, low-clinker and clinker-free binders are actively being developed to decarbonize concrete while preserving structural performance.

Alkali-activated materials (AAMs) constitute a broad class of binders produced by activating aluminosilicate and/or calcium-rich precursors with alkaline solutions. Depending on precursor chemistry (particularly calcium content), the reaction products may include N-A-S-H and/or C-(A)-S-H type gels, which can deliver mechanical performance comparable to OPC in many applications [9,10,11,12,13]. In such systems, the binder network forms through dissolution and polycondensation reactions involving Si and Al species, commonly activated using sodium hydroxide and/or sodium silicate solutions, leading to hardened products with mechanical performance comparable to OPC in many cases [14,15,16,17]. Ground granulated blast-furnace slag (GGBFS) is especially relevant because it can promote ambient curing and the formation of C-(A)-S-H type reaction products, enabling structural-grade alkali-activated concretes [17].

Within this framework, diatomaceous earth (DE) offers an underexploited resource for low-clinker binders. DE (kieselguhr) is a silica-rich material derived from diatom frustules and is available both as a natural mineral and as a recycled industrial residue from filtration processes (e.g., wine and other beverages). Its high SiO_2_ content and micro-porous structure make it a potentially valuable reactive or filler component in alkali-activated binders, particularly when combined with calcium-rich precursors such as GGBFS. However, the role of DE is not universal: its reactivity depends on its origin, mineralogy/crystallinity, fineness, and the alkalinity and chemistry of the activating solution. Consequently, mechanical performance reported for DE-containing AAMs can be inconsistent across studies, and experimental evidence at structural scale remains limited.

Beyond compressive strength, structural implementation requires reliable information on elastic modulus, bond performance, and member-level response. Previous studies generally report that alkali-activated concretes can reach compressive strengths comparable to OPC concretes [18,19], although elastic modulus may be lower in several formulations [20]. Concerning steel-concrete bond, some works report higher bond capacity than OPC at comparable strength levels-potentially linked to tensile properties and interfacial densification-whereas others find similar bond levels and emphasize the correlation with tensile strength [21,22,23,24,25]. At member level, reinforced alkali-activated beams are often reported to exhibit overall flexural behavior comparable to OPC beams, with differences in stiffness, cracking pattern, and deformation capacity influenced by modulus, bond, and reinforcement ratio [26,27,28,29,30,31,32,33,34,35,36].

Despite the growing body of work on alkali-activated/alkali-activated concretes, the literature shows non-uniform and sometimes contradictory evidence regarding structural performance indicators, particularly steel–concrete bond. While several studies report bond strengths comparable to, or even exceeding, OPC concretes at similar compressive strength levels, other works find no systematic improvement and emphasize that bond response is strongly dependent on factors beyond compressive strength, such as tensile resistance and splitting propensity, activator chemistry, precursor composition (e.g., slag-vs fly-ash-dominated systems), curing regime, and specimen/test configuration (embedment length, cover, confinement, and bar rib geometry). These discrepancies suggest that alkali-activated concrete bond performance cannot be generalized without specifying mixture design and testing conditions. Accordingly, a combined evaluation of material properties, bond stress-slip response, and member-level behavior is needed to clarify how mixture-dependent matrix characteristics translate into reinforced structural performance-providing the basis for the present experimental program.

The present study investigates alkali-activated concretes incorporating natural diatomaceous earth (M2 and M3) and recycled diatomaceous residues from industrial filtration (V6–V7), combined with GGBFS, and benchmarks them against a conventional OPC concrete. Mixtures were designed using slag-only binder (R1), slag + natural diatoms (R6–R7), slag + wine-derived diatoms (RV6–RV7), and slag + recycled diatoms (R6M3–R7M3). The experimental program evaluates mechanical properties (compressive, tensile, flexural strengths and modulus of elasticity), steel-concrete bond behavior through pull-out response, and structural performance via reinforced beam tests under four-point bending. By integrating material-level and structural-level results for diatom-based alkali-activated concretes, the study provides evidence for the feasibility and limitations of recycled DE as an alternative binder component for structural concrete applications.

## 2. Experimental Program

The experimental program focused on the production of alkali-activated concretes incorporating industrial waste as raw materials. This study investigates the use of different types of natural diatomaceous earth (M2 and M3) as precursors, along with recycled diatomaceous earth derived from industrial waste in the wine filtration process (V) [37]. These diatomaceous materials were combined with ground granulated blast-furnace slag (GGBS). The natural diatoms were crushed and milled to serve as an aluminosilicate source.

Diatomite (kieselguhr) is a siliceous sedimentary rock composed predominantly of the fossilized remains of diatoms (unicellular aquatic organisms). Over time, these remains accumulate and consolidate into a diatomaceous mud or sludge rich in biogenic silica derived from the diatom frustules. Diatomite has a high melting point (≈1500 °C) and typically contains more SiO_2_ [38].

Several studies have investigated diatomaceous earth as a partial substitute for fly ash and as an additive in fly ash-based alkali-activated formulations. In addition, diatomaceous earth has been used in lightweight concrete, in the industrial-scale production of alkali-activated binders, and as a cementitious additive in different mixtures. Other studies have also examined the combined use of rice husk ash and diatomaceous earth in alkali-activated systems.

Wine and beer filtration diatomaceous residues (kieselguhr) are particularly attractive for alkali-activated systems because they typically consist of silica-rich diatom frustules with high internal porosity and specific surface area, which can enhance alkaline dissolution and provide abundant reactive silicate species. In addition, these residues may contain minor carbonate and organic fractions (often reflected by loss on ignition, LOI), and trace impurities inherited from the filtration process; such features can affect the effective alkalinity demand and reaction kinetics. When combined with calcium-rich precursors such as GGBFS, the reactive silica supplied by these residues can contribute to the formation of hybrid binding gels and improved early-age hardening, supporting their potential as sustainable components in structural alkali-activated concrete.

### 2.1. Materials Data and Experimental Setups

#### 2.1.1. Raw Materials Description

**Figure 1:**
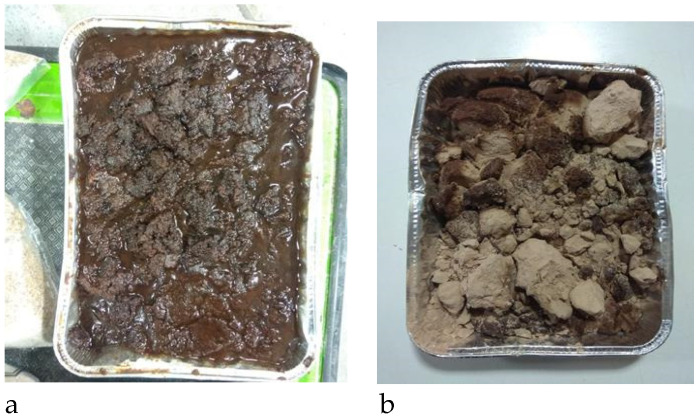
Dried recycled diatomite residues after oven treatment at 100 °C for 48 h: (**a**) wine filtrate (**b**) oil filtrate.

During this phase, the raw materials were characterized to determine their physical and mechanical properties. The ordinary Portland cement (OPC) used in this study was CEM I 42.5R, manufactured in accordance with the Spanish standards RC-16 and the European standars UNE-EN 197-1, and supplied by Cementos La Cruz, S.L., Abanilla, Spain. As raw materials, by-products from different sources were employed. Two types of diatomaceous earth were used: natural diatoms (M2 and M3) and dried recycled wine diatoms (V6 and V7). To promote alkali activation and improve compressive strength, ground granulated blast-furnace slag (GGBS) was incorporated, with its dosage adjusted to maintain the target Si/Al ratio in the binder. Prior to mixing, the recycled diatomite samples were oven-dried at 100 °C for 48 h to remove residual moisture, whereas the natural diatoms were supplied already dried and were therefore not re-dried (Figure 1).

**Figure 2:**
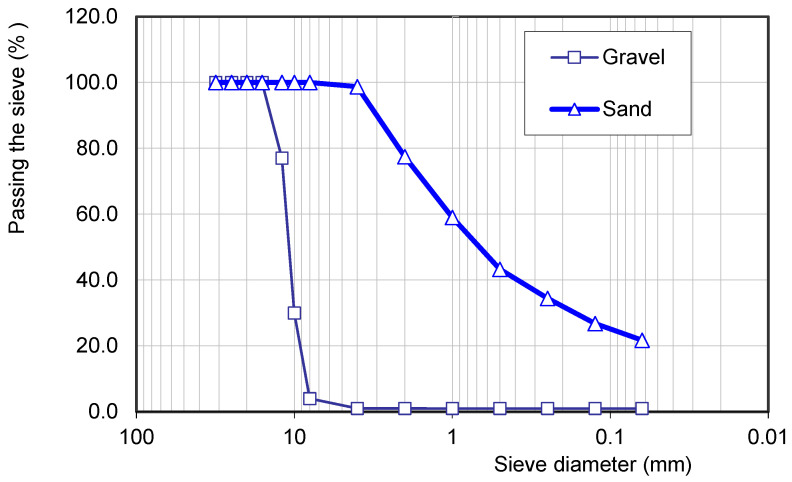
Grading curve of aggregates.

Crushed limestone aggregates were used as the coarse aggregate, featuring a maximum particle size of 12 mm (5–12 mm), a specific gravity of 2.63 g/cm^3^, and a water absorption rate of 1.10%. Fine aggregate consisted of sand with a specific gravity of 2.55 g/cm^3^ and a water absorption rate of 0.9%. The particle size distribution is illustrated in Figure 2.

Sodium hydroxide was employed as the activator for the diatomaceous earth and ground granulated blast-furnace slag precursors. The sodium hydroxide (NaOH) was in pellet form (99% purity). NaOH pellets were dissolved in the mixing water under stirring until complete dissolution. Because dissolution is exothermic, the solution was prepared 24 h prior to mixing and kept at room temperature to cool and stabilize before use. For reproducibility, the activator dosage is reported as Na_2_O-equivalent content (4–10% Na_2_O in the mortar optimization campaign) and as the corresponding NaOH molarity of the prepared solution (as shown in Table 7 where the results are presented in Section 2.2.1, together with the criteria used to optimize the mix design), which ranged from approximately 2.0 to 5.6 M. The Na_2_O-equivalent associated with NaOH follows 2NaOH → Na_2_O + H_2_O, hence m(Na_2_O) ≈ 0.775 m(NaOH). Higher molarities are reported in the literature and may increase the risk of efflorescence; therefore, the present study focused on the above range to balance reactivity and durability considerations [39,40].

An extensive experimental campaign was conducted, comprising particle size analysis, mineralogical analysis via X-ray diffraction (XRD) to quantify the amorphous material content, X-ray fluorescence (XRF) analysis to determine the chemical composition of the materials, and thermogravimetric analysis (Table 1).

The leachate results (Table 2) show elevated alkalinity (high pH), which is expected for both OPC and alkali-activated binders due to the presence of alkaline pore solutions and soluble alkali species. From a practical standpoint, such high pH values may impose constraints in early-age stages and in applications where the material may be in direct contact with water (e.g., runoff during curing, wash water management, or storage/processing prior to carbonation), since alkaline effluents can require controlled handling and, where necessary, neutralization. For commercial application, environmental safety and regulatory compliance should therefore be evaluated using the appropriate standardized leaching protocols and acceptance criteria relevant to the intended use scenario. The present leachate results are reported as a screening assessment; additional compliance-oriented testing under representative exposure conditions is recommended before large-scale implementation.

On the other hand, the leachate results (Table 2) indicate that the investigated alkali-activated mixtures do not exhibit abnormal release of the analyzed species under the applied test conditions, suggesting satisfactory environmental safety in terms of contaminant immobilization for the studied compositions. In practice, leaching performance is a key screening indicator for the use of industrial by-products and residues in cementitious matrices; however, it does not by itself quantify the overall environmental footprint of the material. Nevertheless, these results support the potential for improved environmental performance through the valorization of industrial residues, while acknowledging that a dedicated life-cycle assessment (LCA) is required to quantify the net environmental benefits.

#### 2.1.2. Fineness and Laser Diffraction for Particle Size Analysis of Fine Particles

The fineness of the ground slag was determined using the Blaine air permeability method, following the UNE EN 196-6:2010 standard (Table 3).

The porous texture of the diatomaceous earth is characterized by nitrogen adsorption isotherms at 77 K. In this characterization, key parameters such as specific surface area, pore volume, and pore size distribution were measured. The nitrogen adsorption isotherm provided pore size information in the range of 3.5 nm to 400 nm (Table 4). This analysis was conducted by the Technical Services Department of the University of Alicante, utilizing an AUTOSORB-6 volumetric gas adsorption system and an AUTOSORB DEGASSER, both manufactured by QUANTACHROME INSTRUMENTS, Boynton Beach, FL, USA.

In general, smaller particle sizes tend to exhibit higher reactivity due to their greater specific surface area, as seen with ground granulated blast-furnace slag. Specifically, a larger reactive surface area enhances hydration speed and efficiency. A higher specific surface area accelerates reactions because the energy required to initiate the reaction decreases, owing to the proportionally greater number of active sites on particles with an elevated specific surface area.

**Figure 3:**
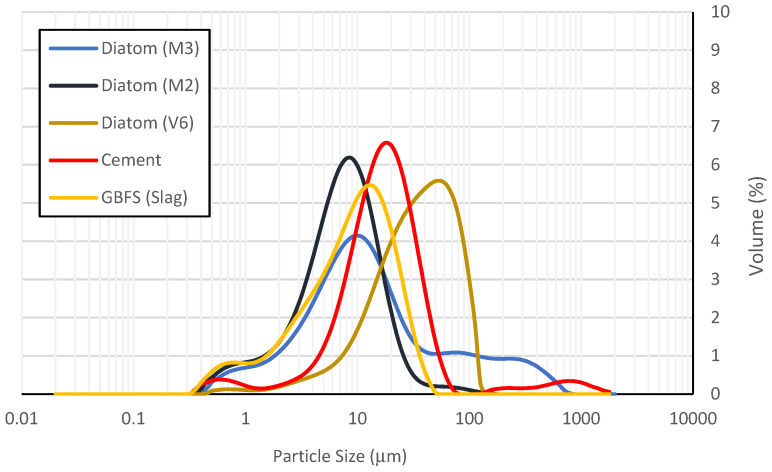
Laser particle size of the diatomaceous earth, slag and cement used.

To assess the distribution of fine particle sizes, a Mastersizer 2000 particle size analyzer by Malvern Instruments Ltd., Malvern, UK, was employed. The natural diatom particles exhibited sizes ranging from 0.4 to 130 µm, with a peak presence around 9 µm, displaying an almost unimodal distribution (Figure 3). Besides the type of precursors used, the fineness of diatomaceous earth particles is another factor influencing the performance of alkali-activated concretes. Finer particles improve weak Van der Waals adhesion forces and potentially alter the crystalline structure and reactivity of the particles.

#### 2.1.3. X-Ray Diffraction

Mineralogical analyses were conducted using X-ray diffraction (XRD). The equipment was operated with CuKθ radiation (1.542 Å) under the following conditions for all analyses: a scan range of 10 to 80° 2θ, with a step size of 0.03° 2θ and an exposure time of 3 s per step. The samples were dried at 110 °C, ground to particle sizes below 50 μm, and mounted on aluminum sample holders. Mineral phase identification was performed using the JCPDS (Joint Committee on Powder Diffraction Standards) database.

**Figure 4:**
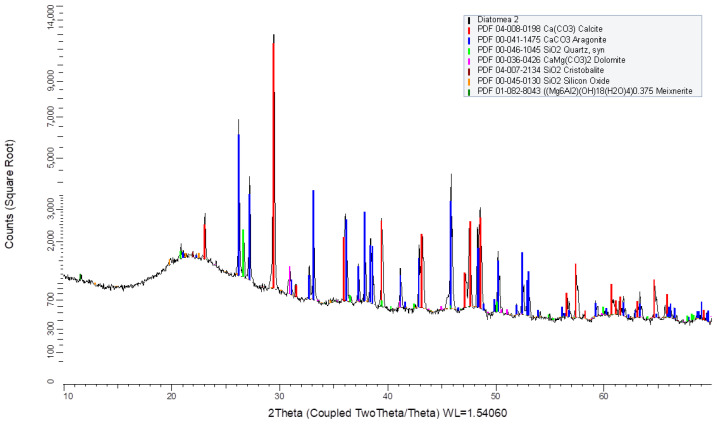
XRD pattern of Diatom M2.

**Figure 5:**
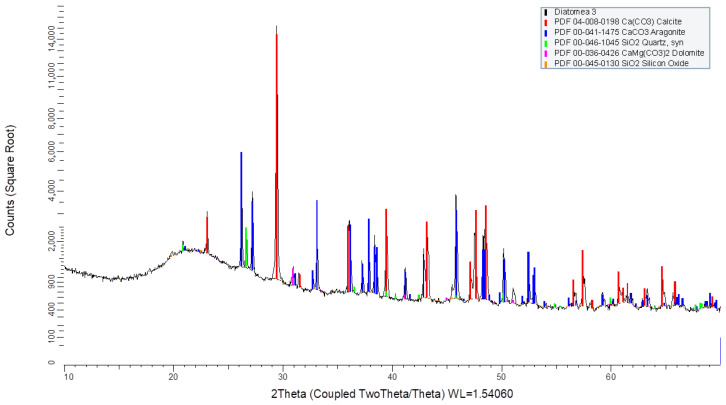
XRD pattern of Diatom M3.

**Figure 6:**
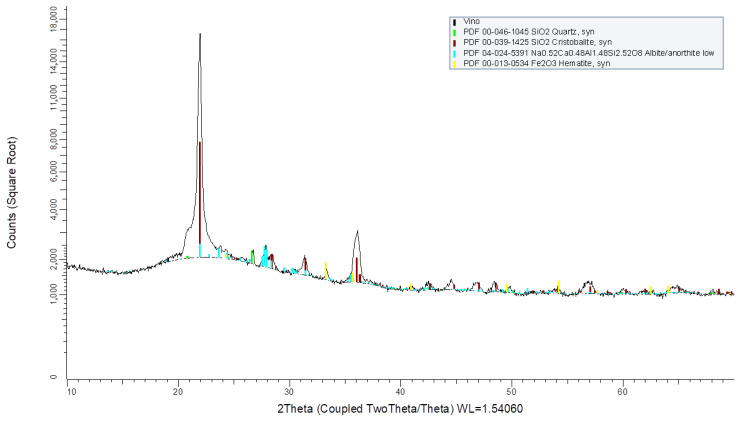
XRD pattern of Diatom V6.

The XRD patterns (Figure 4, Figure 5 and Figure 6) revealed that the predominant crystalline phases in all diatomaceous samples were quartz and cristobalite, both distinct forms of silica. Minor quantities of other crystalline phases were also detected, including tridymite (SiO_2_) in CD, anorthite (CaAl_2_Si_2_O_8_) in BD, and calcite (CaCO_3_). The high crystallinity of these materials may contribute to their low reactivity, rendering them less effective in the polymerization/gelation process and leading to reduced compressive strengths.

The chemical composition of the diatomaceous materials, expressed as oxides, was determined by X-ray fluorescence (XRF), and the results are summarized in Table 5. For both the natural (M2 and M3) and recycled diatoms, the high SiO_2_ content is particularly noteworthy, except for the wine diatoms, which exhibited lower SiO_2_. Significant amounts of Al_2_O_3_ and Fe_2_O_3_ were also detected. The sum of SiO_2_ + Al_2_O_3_ + Fe_2_O_3_ exceeded 80% in all diatomaceous samples, indicating their substantial potential for alkali-activated synthesis. The primary components of the diatoms and slag were SiO_2_ and Al_2_O_3_, while the slag additionally contained large amounts of CaO.

Both natural and recycled diatoms exhibited relatively similar oxide compositions, with a high SiO_2_ content (51–70%) and a lower Al_2_O_3_ content (11–12%). Minor amounts of MgO and CaO were also detected. This composition suggests an adequate availability of Si and Al species that can be effectively dissolved and re-polymerized during alkaline activation, promoting the formation of Si-O-Al bonds. In contrast, ground granulated blast-furnace slag (GGBS) showed nearly comparable contents of SiO_2_ and CaO (31–35%) and a lower Al_2_O_3_ content (~12%).

### 2.2. Mix Proportions Used for Alkali-Activated Concrete

#### 2.2.1. Optimizing the Alkaline Precursor Activation Process

The optimization study aimed at identifying the most effective activating agent and concentration in the alkaline activation process focused on the mechanical properties of the resulting mortars. Mortars were prepared with a precursor-to-aggregate ratio of 1:2, using standardized siliceous sand as the aggregate.

Initially, the activating solution with NaOH as the activator used a solution-to-diatom ratio of 0.4 and four different Na_2_O concentrations: 4%, 6%, 8%, and 10%. Similarly to the case of WG, the solution-to-diatom ratio was increased to 0.7 to achieve a workable mixture. The resulting ratios and quantities are presented in Table 6, with an initial material weight of 675 g (Table 6 and Table 7).

After the first experimental campaign with concrete and based on the studies by Palacios and Puertas [38], it was established that the mixing time should be 10 min from the moment sodium hydroxide is introduced into the mixer, as this has a significant impact on the development of mechanical strength in these activated systems. They note that extending the mixing time (up to 30 min) could enhance strength by up to 11% and reduce system permeability due to a decrease in the volume of small pores.

#### 2.2.2. Mix Proportions

**Figure 7:**
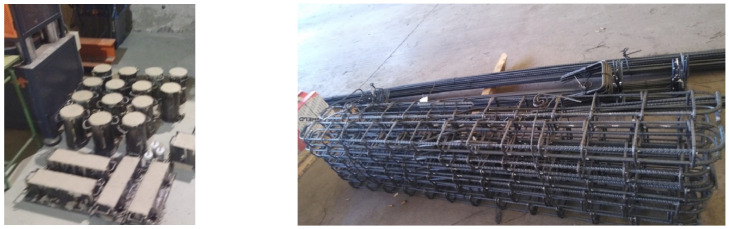
Samples of the specimens made and of the reinforcement to execute the beams.

After obtaining the characterization results for all materials and analyzing the preliminary mix proportions, a series of final dosages were determined based on the findings from the mortar tests and subsequently evaluated in the laboratory (Figure 7). The mix proportions are shown in Table 8.

### 2.3. Tests Conducted for the Alkali-Activated Concrete

To characterize the hardened alkali-activated concretes at both material and structural scales, the experimental program comprised compressive strength, splitting tensile strength, flexural strength, static modulus of elasticity, steel-to-concrete bond (pull-out) and reinforced beam tests. The selection of tests and reporting format follows the corresponding European standards (UNE-EN) and widely adopted RILEM recommendations for bond characterization.

Specimen preparation, demolding and curing were performed according to UNE-EN 12390-2. All specimens were demolded after approximately 24 h and cured under indoor laboratory conditions (T ≈ 20–23 °C; RH ≈ 45–60%) until the specified testing age. These conditions were kept constant for all mixtures to ensure comparability. While alkali-activated binders are often reported to be less sensitive than OPC systems to moderate indoor fluctuations, temperature and humidity still influence reaction kinetics and early-age strength development; therefore, the curing environment is reported explicitly.

Mechanical testing was conducted using a servo-controlled universal testing machine with a calibrated load cell and displacement measurement. Compressive strength was determined in accordance with UNE-EN 12390-3 (with ASTM C39 as reference), splitting tensile strength in accordance with UNE-EN 12390-6/ISO 4108 (with ASTM C496 as reference), flexural strength in accordance with UNE-EN 12390-5 (with ASTM C78 as reference) and the static modulus of elasticity in accordance with UNE-EN 12390-13 (with ASTM C469 as reference). Specimen geometries, loading configurations and loading rates were selected to comply with the requirements of the cited standards and to ensure comparability with the available literature.

Unless otherwise stated in the corresponding subsections, n = 3 replicate specimens per mixture were tested for each material-scale property (compressive strength, splitting tensile strength, flexural strength and modulus of elasticity) and mean values are reported; dispersion indicators are provided where relevant. Bond stress-slip curves were obtained from n = 3 pull-out specimens per mixture, with bond stresses reported at specified slip levels and with failure mode classification (pull-out versus splitting). Reinforced beam tests were performed on one specimen per mixture; accordingly, beam results are interpreted as comparative member-level evidence rather than a statistical characterization.

From an application perspective, mixture selection should also account for practical constraints, including workability/castability, activator handling and quality control of solution preparation, and the implications of alkaline leachate for early-age wash water/runoff management. Long-term durability under representative exposure conditions is identified as a key next step prior to large-scale implementation.

### 2.4. Compressive Strength

**Figure 8:**
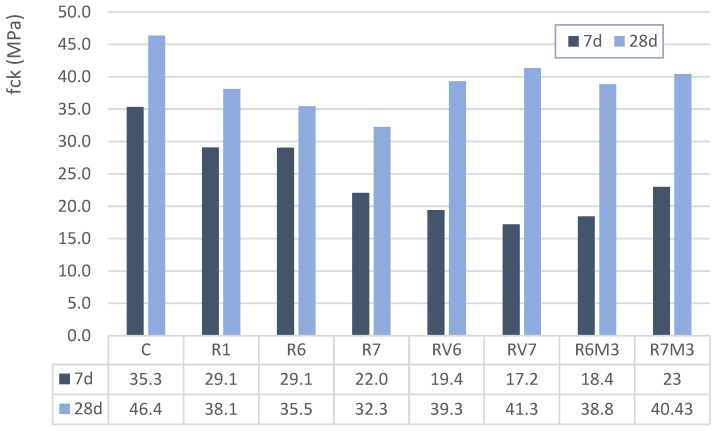
Compressive strength at 7 and 28 days.

Three specimens were tested for each mixture at 7, 28, and 90 days. Specimen casting, curing and storage followed UNE-EN 12390-2, and compressive strength testing followed UNE-EN 12390-3. The compressive strength of the alkali-activated concretes cured for 7 and 28 days is presented in Figure 8. alkali-activated materials composed solely of slag (R1) demonstrated compressive strength comparable to that of concretes incorporating M2 diatoms [40]. At 7 and 28 days, R1 concretes achieved compressive strengths of 29.1 MPa and 38.1 MPa, respectively, compared to the average strengths of 25.5 MPa and 33.9 MPa for mixtures R6 and R7. This indicates that slag-only alkali-activated materials exhibited slightly higher compressive strength values, with improvements of 13.7% and 12.58% at 7 and 28 days, respectively.

The results obtained with wine-derived diatoms and M3 diatoms are noteworthy, with the RV mixtures demonstrating an average strength of 18.3 MPa at 7 days and 40.3 MPa at 28 days, and the M3 mixtures exhibiting an average strength of 20.7 MPa at 7 days and 39.6 MPa at 28 days. This indicates an almost twofold increase in strength between 7 and 28 days. These two alkali-activated mixtures achieved the highest compressive strengths, closely approaching those of the reference concrete.

The comparatively high compressive strengths obtained for the M3-based mixtures (R6M3 and R7M3) cannot be explained by fineness alone. In alkali-activated systems, strength development is governed by the dissolution of reactive Si and Al species and subsequent gel formation (Si-O-Al bonds), which is strongly influenced by precursor chemistry and mineralogy. From XRF results (Table 5), natural diatoms (M2 and M3) present high silica contents and relatively low alumina contents, implying a high SiO_2_/Al_2_O_3_ ratio. Such chemistry favors the availability of silica for polymeric network formation, while the presence of slag supplies Ca and Al that can promote the formation of hybrid binding gels (C-(A)-S-H and/or N-(C)-A-S-H type products), improving early and later-age strength.

Mineralogical analysis by XRD (Figure 4, Figure 5 and Figure 6) indicates that the diatomaceous materials are dominated by crystalline silica phases (quartz and cristobalite), with minor additional phases. Because high crystallinity generally reduces dissolution rate in alkaline media, the effective reactivity is expected to be controlled primarily by the amorphous fraction and by the interaction between the diatomaceous component and slag-derived species. Within this context, M3 exhibits higher fineness and BET surface area than M2 (Table 2 and Table 3), which increases the effective reactive surface and can enhance dissolution kinetics and gel formation despite the presence of crystalline silica phases. Therefore, the strength gain observed for M3 mixtures is consistent with a combined effect of favorable Si-Al chemistry, higher available reactive surface area, and synergistic reaction with GGBFS in alkaline activation.

This behavior could be attributed to the lower densification of these specimens and the reduced formation of alkali-activated binder gel. In addition, NaOH incorporation increases the (Na + K)/Si ratio, which may influence strength development in alkali-activated materials. This ratio is closely associated with the extent of alkali activation, since sufficient alkalinity is required to promote the dissolution of aluminosilicate phases in the raw materials (slag and diatoms). A higher alkali concentration generally accelerates the dissolution of solid precursor particles, thereby enhancing alkali-activated binder gel formation and, consequently, compressive strength. According to the X-ray fluorescence results (Table 5), the highest SiO_2_ content was observed in the wine diatoms, which is consistent with the trends discussed above.

Nevertheless, the average compressive strength values at 28 days indicate that these materials can be effectively used in conventional structural applications, demonstrating that residual diatomaceous earth can be successfully utilized as a silica source in alkali-activated systems. Although all residues exhibited lower reactivity compared to the original Portland cement, the findings suggest promising new alternatives for the reuse and valorization of these by-products. Residual diatomaceous earth achieved similar results (RV7) and even superior outcomes (R6 M3 and R7M3) compared to commercial diatomite (C).

It is essential to recognize that the progression of strength development varies significantly depending on the precursor used in alkali-activated production, warranting further investigation for each material. Notably, while strength gains in Portland cement concrete typically range from 15% to 30%, alkali-activated materials have shown variations of up to 140%, attributed to the slower alkali-activation processes.

Beyond particle fineness, the significant variation in strength development among mixtures is consistent with the mixture-dependent kinetics of alkali activation. In the present materials, XRF/LOI indicates silica-rich diatomaceous precursors, while XRD reveals predominance of crystalline silica phases (e.g., quartz/cristobalite), which generally dissolve more slowly in alkaline media than amorphous phases. Therefore, the effective contribution of diatoms to alkali-activation is expected to be governed by the amorphous/reactive fraction and the available reactive surface, as well as by the interaction with slag-derived species that promote ambient hardening. This provides a plausible explanation for the observed differences in strength evolution across diatom sources and mixtures.

### 2.5. Tensile Strength

Indirect tensile strength and flexural strength tests were conducted at 28 days. The figure displays the tensile strength of the studied mixtures at the age of 28 days.

#### 2.5.1. Indirect Tensile Strength

**Figure 9:**
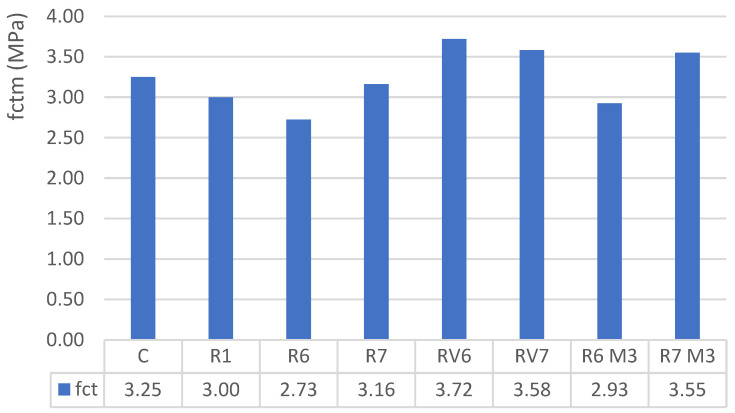
Average results of the indirect tensile test.

The indirect tensile strength test is a widely adopted procedure internationally, governed by standards UNE-EN 12390-6 and ISO 4108. The results indicate that in the tested mixtures, alkali-activated materials achieved slightly higher tensile strength compared to a standard Portland cement concrete (Figure 9), but within the ranges established by the codes, reaching an average tensile strength of 8.5% of their compressive strength, compared to 7% in the reference C concrete.

**Figure 10:**
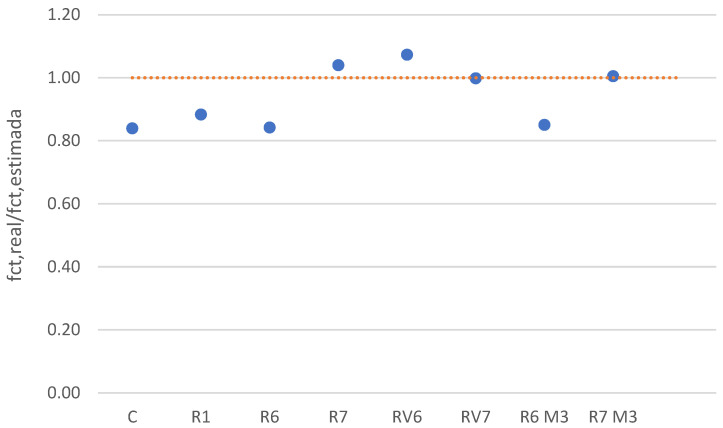
Comparison between expected tensile strength by achieved compressive strength and experimental tensile strength.

When comparing these values with those obtained by correlating compressive strength with the normative average tensile strength (fctk = 0.30·(fc)^2/3^), it is observed that the differences between the estimated and normative values range from 4% to 16%. Given that the variability inherent in the testing process is approximately 18%, these values are considered acceptable and within the normal range (Figure 10). Therefore, the relationship between compressive strength and tensile strength falls within the typical patterns observed in conventional concrete.

#### 2.5.2. Flexural Strength

The determination of flexural strength (fcf) is standardized under UNE-EN 12390-5. This test was performed at 28 days on two prismatic specimens measuring 100 × 100 × 400 mm for each of the 10 mix designs.

**Figure 11:**
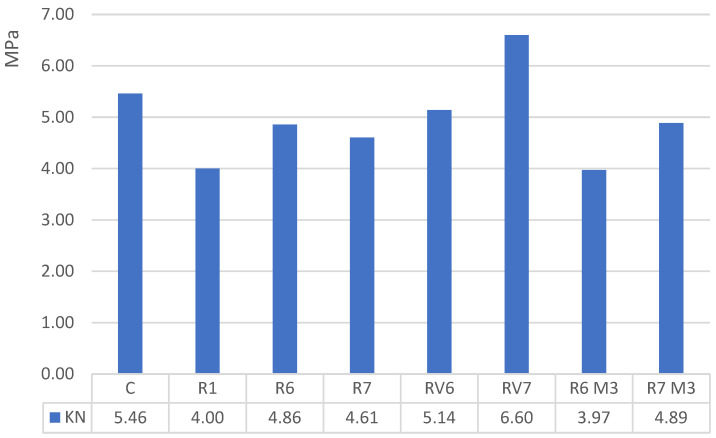
Flexural Strength in MPa.

The flexural strength obtained in the alkali-activated materials is lower compared to OPC-based concrete, except in the RV7 mixture (Figure 11). Nevertheless, this improvement is relatively slight, as the average flexural strength of the RV6 and RV7 mixtures is 5.87 MPa, compared to 5.46 MPa in the reference C mixture, representing only a 7.45% increase. The R6 and R7 mixtures, prepared with M2 and M3 diatoms, exhibit flexural strengths that are 13.35% and 18.87% lower than those of the reference concrete. While the diatoms may appear chemically similar to GGBS, their composition differs. The combination of GGBS and V7 diatoms stands out due to its relatively balanced levels of silica, calcium, and alumina.

However, if the results are parameterized by dividing them by the obtained tensile strength, it is observed that the values range between 10% and 16% of the tensile strength. Given that the test exhibits variability of up to 20%, these values are considered to fall within the expected range for concrete.

### 2.6. Elastic Modulus

The slope of the stress–strain relationship in concrete depends, among other factors, on internal microcracking [41]. The elastic modulus is strongly influenced by the modulus of the aggregate, as well as its shape and texture [42]. The modulus of elasticity is directly proportional to compressive strength and tends to increase as compressive strength rises. Consequently, as compressive strength increases, concrete tends to lose “ductility” and become more brittle.

The measurement was performed by conducting three loading/unloading cycles. The elastic modulus was determined based on the loading slope of the stress–strain curve. The test was carried out in accordance with the UNE-EN 12390-13:2014 standard. The following table presents the average values of the static modulus of elasticity at 28 days.

**Figure 12:**
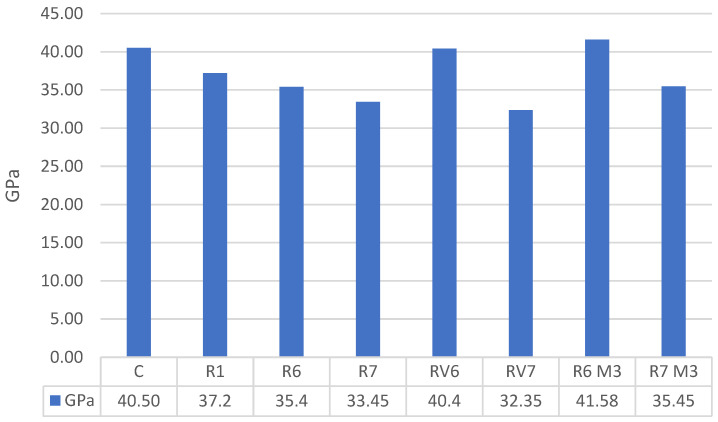
Strain modulus in GPa.

The results indicate (Figure 12) that the modulus of elasticity in the R1 mixture is comparable to that of the OPC concrete mixture, whereas the alkali-activated materials made with M2 diatomite exhibit lower values. The mixtures made with wine-derived diatoms display varying results, with the RV6 mixture achieving significantly higher strength, while the RV7 mixture shows slightly lower values, but similar to the R6 and R7 mixtures, underscoring the sensitivity of the polymerization process. The RV6 mixture, consistent with the results from indirect tensile strength tests, exhibits the highest modulus of elasticity, along with the R6M3 mixture. Despite these differences, the variation in results relative to the mean remains below 13%.

**Figure 13:**
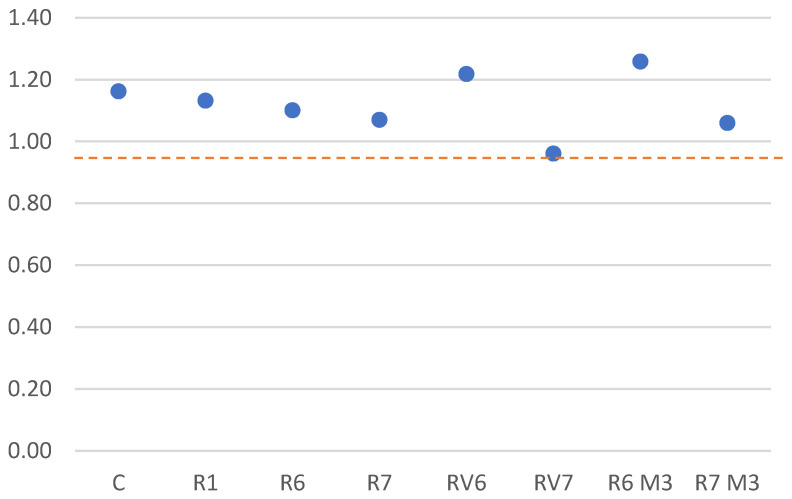
Comparison between the expected deformation modulus and the experimentally measured deformation modulus.

When comparing the estimated values from EC-2, based on compressive strength (Ec = 22·(fcm/10)^0.3^ in GPa), it is evident that the obtained values lie within the safe range, except for the RV7 mixture (Figure 13).

The observation that alkali-activated mixtures can exhibit a slightly lower elastic modulus while maintaining comparable (or even improved) tensile/flexural performance is not necessarily contradictory. The elastic modulus is primarily controlled by the bulk matrix stiffness and porosity, whereas tensile/flexural response is governed by cracking and fracture processes. Therefore, mixtures with similar strength but different pore structure and gel assemblage may show a reduced modulus without proportional changes in tensile or flexural capacity.

### 2.7. Bond Strength

**Figure 14:**
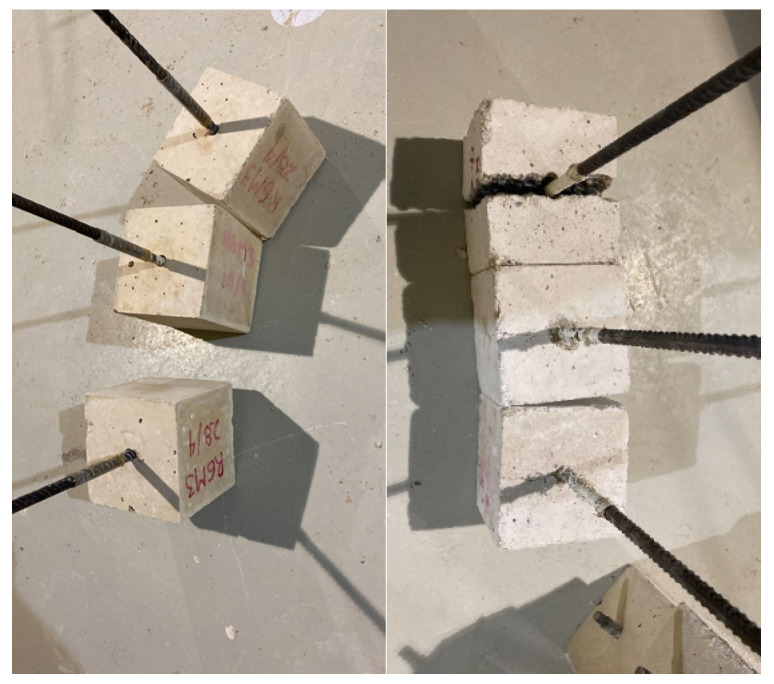
Failure due to pullout of the bar (**left**) and failure due to specimen breakage or splitting of the concrete (**right**).

In the pull-out tests, the load and slip of the embedded steel bar were measured (Figure 14). The test configuration and loading procedure were established following the RILEM/CEB/FIP recommendation for pull-out tests (RC6) [43], using ribbed steel bars with a diameter of 12 mm embedded in 150 mm cubic specimens without transverse confinement. The embedment depth was set to five bar diameters (5ϕ), while the remaining bar length was covered with a rubber sleeve to eliminate bond outside the intended bonded zone. According to RC6, the loading rate depends on the bar diameter and was calculated using the expression (N/s). For the 12 mm bars, a loading rate of 72 N/s was applied.

**Figure 15:**
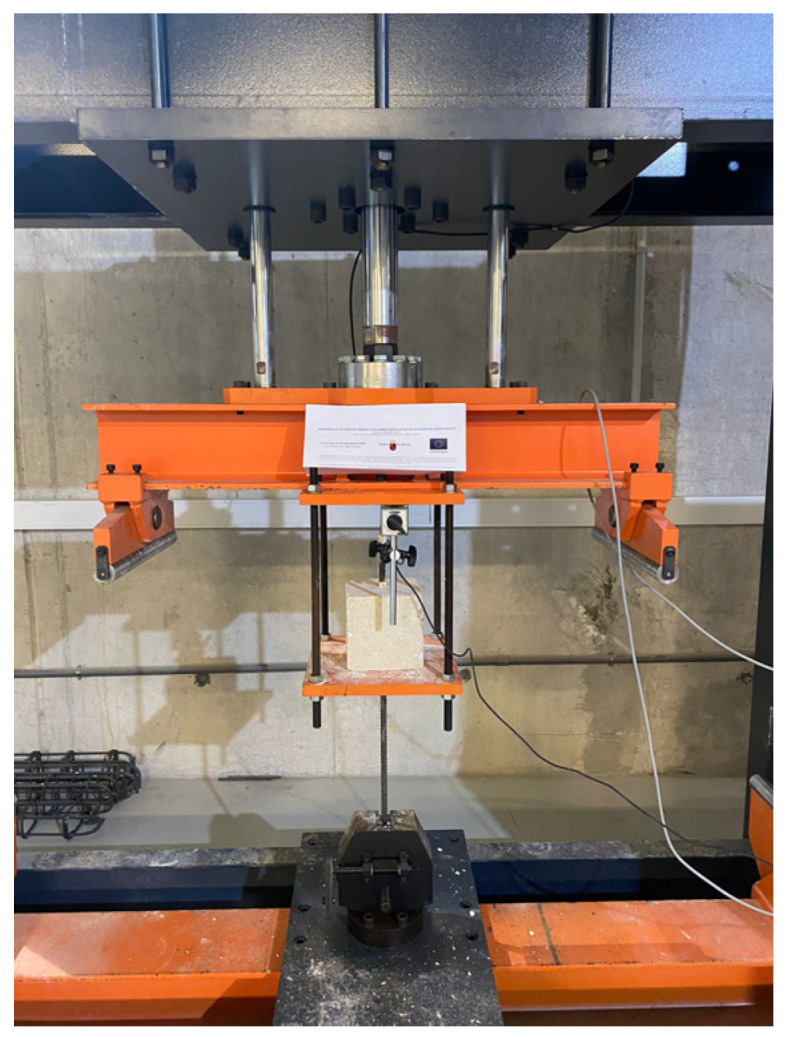
Pullout or adhesion test specimen testing gantry.

The test was terminated when failure occurred due to pull-out, cover splitting, or bar rupture (Figure 15). The ultimate bond stress (τ_bu_) is computed by dividing the applied load by the steel–concrete contact surface area:

$$\tau_{bu}=\frac{P_{u}}{l_{s}\pi \phi }$$

where

*P_u_*—maximum or ultimate load prior to failure,

*l_s_*—bond length, and

ϕ—bar diameter.

The expression assumes a constant distribution of stresses along the entire length of the bar, thereby not considering the potential concentration of stresses at the initial ribs, which is insignificant except in the case of very high-strength concretes.

Moreover, it is essential to limit the deformations in the bar to ensure satisfactory in-service performance and to uphold the assumption that, under applied loads, the reinforcement and the surrounding concrete experience the same deformation. For this purpose, the average bond stress (τ_bm_) is employed, calculated as the mean of the stresses corresponding to slips of 0.01 mm, 0.1 mm, and 1 mm.

$$\tau_{bm}=\frac{\tau_{0.01}+\tau_{0.1}+\tau_{1}}{3}$$

**Figure 16:**
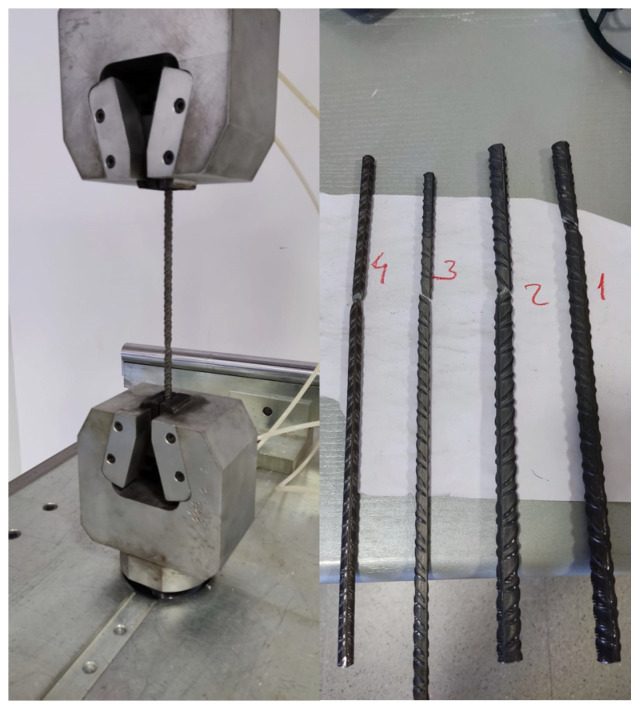
Tests carried out on steel bars; numbers 1–4 indicate the four tests performed for steel characterization.

The steel used in the tests is B500SD. The mechanical properties of the reinforcing bars (yield stress, tensile strength, and elastic modulus) are determined in accordance with ISO 15630-1. Tests performed on the steel (Figure 16) indicate that the average yield stress of the tested bars is 548.5 MPa and the average tensile strength is 657.35 MPa, complying with the requirements for B500SD steel according to Spanish standards UNE 36065 and UNE 36060.

**Figure 17:**
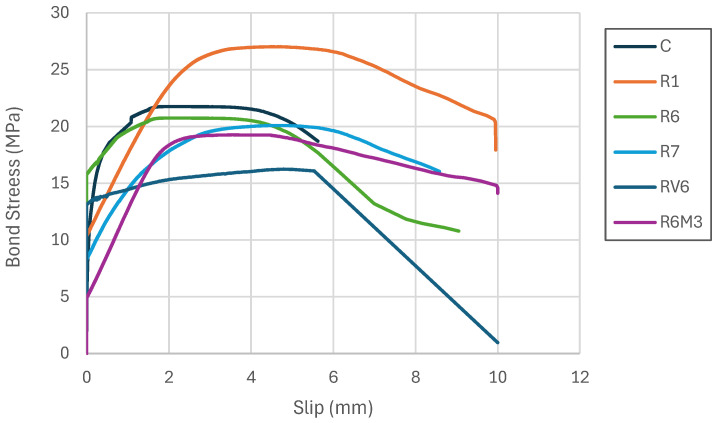
Curves of bond stress-slip (age: 28 days).

The pull-out test results used to assess the bond behavior of alkali-activated concretes are presented below. The bond–slip (τ–s) response is shown in Figure 17. In general, the τ–s curve comprises three stages: an ascending branch, a descending branch, and, in some cases, a residual branch. As stress increases, the response exhibits a steep initial slope, followed by a plateau region around the peak bond stress, and then a gradual post-peak decay as slip increases. At 28 days, all mixtures show good initial stiffness, which is consistent with improved paste–bar interfacial conditions, although the peak bond stress differs among mixtures. The R1 mixture exhibits the highest ultimate bond stress, which can be attributed to the slag content that densifies the interfacial zone without significantly restraining relative slip.

Before analyzing these results, it is important to note that bond stress is strongly influenced by concrete quality, particularly compressive and tensile strengths. Therefore, when comparing mixtures with different strength levels, it is standard practice to normalize bond stress using the square root of the compressive strength, as originally proposed by Orangun et al. [44] and later adopted in design-oriented guidance [27,45,46,47]. In this study, the normalized bond stress was calculated by dividing the measured bond stress by √f_c of the corresponding mixture, with f_c expressed in MPa, consistent with common practice in the literature on alkali-activated materials (Table 9).

Regarding the normalized average bond strength, the R6 mixtures exhibited the highest value (2.89 MPa), followed by RV6 (2.18 MPa). The R6M3 mixture achieved the lowest bond strength (1.25 MPa) (Table 8). Except for R6M3, the results indicate that alkali-activated concretes develop average bond strengths equal to or higher than those of the reference concrete. This suggests that alkali-activated materials can provide adequate steel–concrete bonding, particularly at slips below 1 mm.

The steel-concrete bonding mechanisms are classified into three types: chemical bond, friction, and mechanical bond. The chemical bond primarily relies on van der Waals forces, meaning that certain alkali-activated materials can modify bond strength due to chemical factors. For instance, the addition of slag and diatoms, as demonstrated by Wang et al., can influence bonding. In OPC, the high SiO_2_ content reacts with Ca(OH)_2_ to generate additional CSH. However, in an alkaline environment, a high Ca^+^ content can lead to the formation of Ca(OH) with OH^−^, which may inhibit hydration and reduce strength.

**Figure 18:**
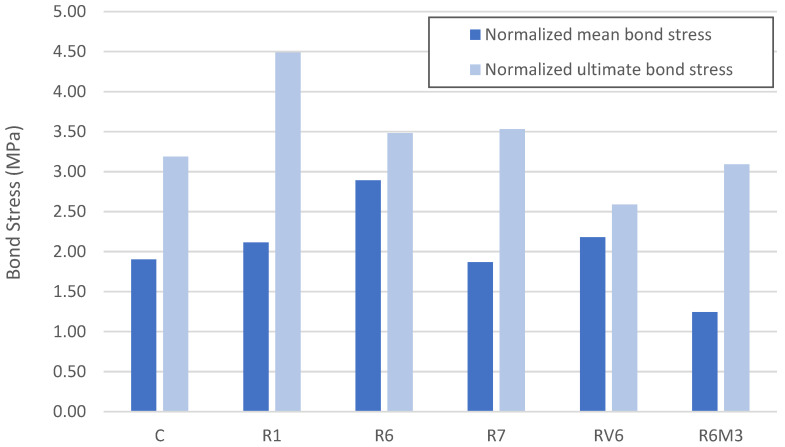
Normalized mean and ultimate Bond stress.

In terms of ultimate bond behavior, the R1 mixture achieves the highest bond strength, which may be attributed to factors related to the alkali activation rate that varies with the addition of diatoms to slag. Nonetheless, the alkali-activated concretes reach an ultimate bond stress equal to or higher than the reference concrete, except for the RV6 mixture (Figure 18).

In terms of failure mode, only the R6 mixture exhibited splitting failure, while the remaining mixes predominantly failed by pull-out (Table 8). Splitting is typically associated with high radial (bursting) stresses around the bar exceeding the tensile capacity of the surrounding concrete cover, especially in unconfined specimens. Therefore, the splitting observed in R6 can be interpreted as the result of its comparatively high bond demand (high bond stresses) combined with limited transverse tensile resistance in the specimen. Conversely, the pull-out failure in R6M3, together with its low bond stresses, indicates that bond capacity at the steel–matrix interface was reached before cover splitting could develop.

Bond performance further highlights this decoupling between stiffness and structural indicators. The bond stress–slip response and failure mode (pull-out versus splitting) suggest that steel–matrix shear transfer depends on mixture-dependent matrix characteristics and tensile resistance, not on compressive strength alone. In unconfined pull-out configurations, high bond demand can trigger splitting when radial stresses exceed the tensile capacity of the surrounding cover, while lower bond levels may lead to pull-out failure. Although SEM/EDS of the bar–paste interfacial zone was not conducted, the consistent differences across slip levels and failure modes provide macroscopic evidence that mixture design affects interfacial shear transfer.

### 2.8. Flexural Structural Behaviour of Beams

#### 2.8.1. Results

Based on the previous results, the research sponsors selected the mixtures with the greatest potential for further investigation, aimed at developing a commercial product. Accordingly, the C mixture was used as the reference, the R1 mixture as the slag-only precursor system, the RV6 mixture as the best-performing formulation, and the R6M3 mixture as the comparatively lowest-performing formulation (Table 10). To assess the structural performance of the manufactured concretes, this study produced eight beams using four mixtures: R1, RV6, R6M3, and the reference concrete.

**Figure 19:**
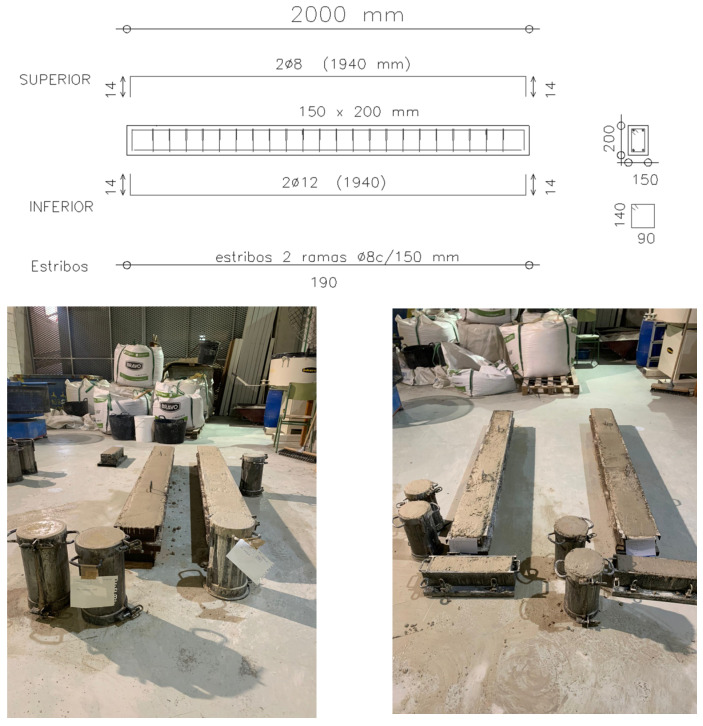
Reinforcement scheme and concreted beams waiting to be tested in the UPCT architectural laboratory.

The beams had a cross-section of 200 × 150 mm^2^ and a total length of 2.00 m. The longitudinal reinforcement consisted of two 8 mm diameter bars in the top layer and two 12 mm diameter bars in the bottom layer, while the transverse reinforcement comprised closed 8 mm diameter stirrups spaced at 150 mm intervals (Figure 19). The theoretical ultimate bending moment, assuming a characteristic compressive strength of 30 MPa (C30/37 according to EN 206/EC 2), was 15.12 kN·m, with an expected neutral-axis depth of 36.74 mm and an ultimate concrete strain of 10 per thousand.

The beams produced have a cross-section of 200 × 150 mm^2^ and a total length of 2.00 m. The reinforcement consists of 2 bars with an 8 mm diameter in the upper section, 2 bars with a 12 mm diameter in the lower section, and transverse reinforcement comprising 8 mm diameter closed stirrups spaced at 150 mm intervals (Figure 19). The theoretical ultimate moment, assuming a concrete with a characteristic compressive strength of 30 MPa (C30/37 according to Eurocode 2) and B500SD steel, is 15.12 kN·m, with an expected neutral axis depth of 36.74 mm, based on a steel failure strain of 10 per thousand.

**Figure 20:**
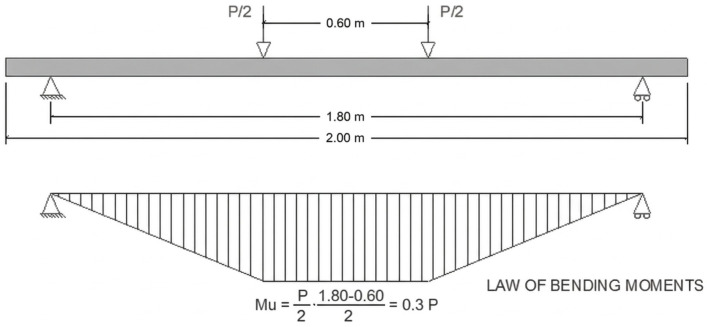
Location of support and pressure points in the press and law of bending moments.

**Figure 21:**
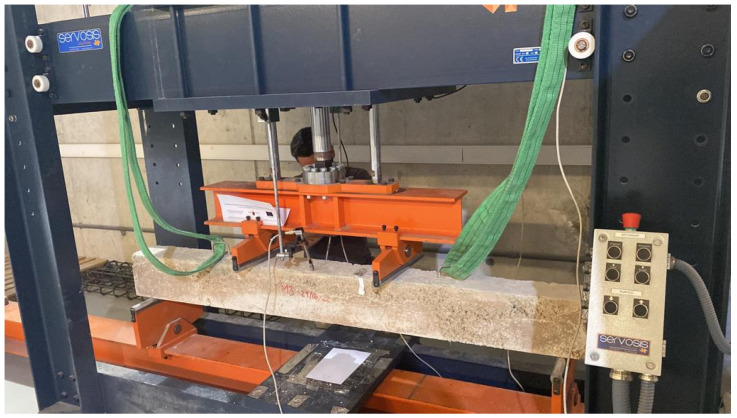
Beam prepared for testing on the gantry with transducers placed on both sides.

Four-point bending tests were conducted in a Servosis test frame, with supports spaced 1800 mm apart and the two load application points separated by 600 mm, creating a constant-moment region between the loads. The load was applied using a hydraulic actuator (2000 kN capacity) and recorded with a calibrated load cell in accordance with ISO 7500-1. All data were recorded at 1 s intervals. The actuator rate was set to 0.05 MPa/s (equivalent stress rate based on the cross-section). Two vertical linear variable displacement transducers (LVDTs) were used to measure midspan deflection (Figure 20 and Figure 21).

**Figure 22:**
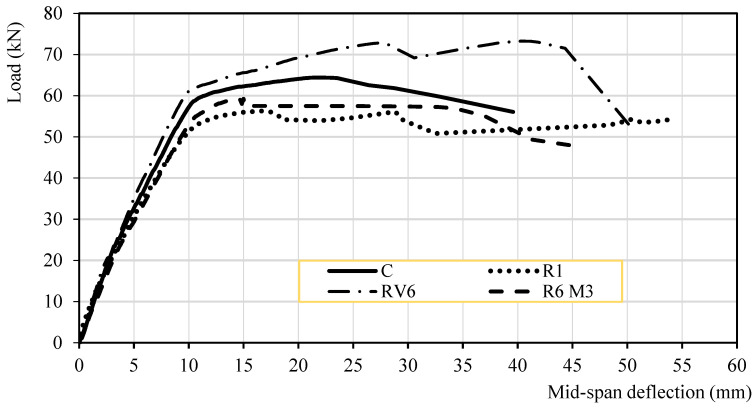
Average tests results of load vs. mid-span deflection.

This study on flexural behaviour focuses on several key factors, including the load–deflection response, yield load, ultimate load, and failure mode. Table 11 and Table 12 summarize the beam-test results, and Figure 22 illustrates the corresponding responses.

#### 2.8.2. Load-Bearing Capacity of Beams

The results obtained from the tests are summarized in Figure 22 and Table 8 and Table 9. The reference beam achieved a higher load-bearing capacity than the R1 and RV6 beams. Nevertheless, the RV6 beam exhibited a relatively high load-bearing capacity, as expected, given its higher compressive and tensile strengths and its enhanced bond characteristics.

Figure 22 and Table 8 and Table 9 summarize the test results. The reference beam achieved a higher load-bearing capacity than the R1 and RV6 beams. Nevertheless, the RV6 beam exhibited a notable capacity, as expected, given its higher compressive and tensile strengths and its enhanced bond characteristics.

Using section equilibrium and the basic assumptions of force equilibrium and strain compatibility (Eurocode 2), an approximate ultimate bending moment for the beam can be calculated. The experimental moments were then compared with the theoretical ultimate moments, considering the compressive and flexural strengths of the concretes (Table 5). Regarding the theoretical moment prediction (Table 10), the RV6 beam exhibited a flexural capacity 26% higher than the theoretical value derived from the concrete mechanical properties. The R6M3 concrete showed a 16% higher capacity than predicted, reaching a value close to that of the reference beam, which achieved a moment 12% higher than the theoretical value. In contrast, the R1 mixture displayed a load-bearing capacity close to the theoretical prediction, which is acceptable but lower than the values reported above.

Furthermore, although previous studies have reported slightly higher deformations in alkali-activated concrete elements, the present results indicate that elastic deformation remains similar, with an average variation of 6.96%. However, the variability of deformation at maximum load was substantially higher, reaching 22%. The ultimate deformation was higher for the RV6 mixture, with mid-span deflections of 27.45 mm at maximum load and 51.96 mm at failure; these values are 25.92% and 31.24% higher, respectively, than those of the reference concrete.

#### 2.8.3. Load-Deflection Curve

The curves representing the relationship between load and mid-span deformation for each specimen, shown in Figure 22, can be interpreted in four phases: (i) pre-cracking, (ii) between cracking and yielding, (iii) from yielding to maximum load, (iv) and from maximum load to failure. During the first phase (up to cracking), all specimens exhibit an approximately linear load–deflection response. Between cracking and yielding, the slope of the curves decreases gradually, indicating a progressive loss of stiffness. Within the elastic load range, mid-span deflection varied from 9.78 to 11.54 mm, and at maximum load it ranged from 15.0 to 27.45 mm; these values increased to an average of 47.59 mm at failure.

**Figure 23:**
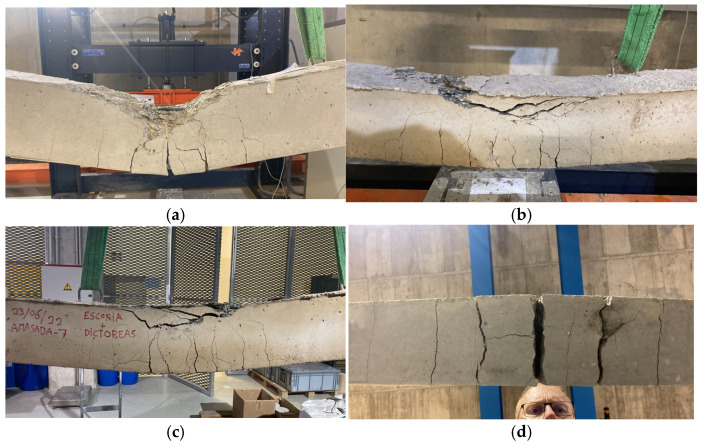
Post-test photographs of the beams tested under four-point bending: (**a**) Beam C (reference concrete); (**b**) Beam R1; (**c**) Beam RV6; (**d**) Beam R6M3. All images correspond to the post-failure condition and document the observed flexural cracking and failure region.

Deformation is a key indicator of ductility. Ductility is a critical property in the plastic behaviour of structures following the formation of plastic hinges. It describes the capacity of an element to continue deforming even when nearing its maximum strength. This deformability not only provides early warning signs of failure but also enables energy absorption, which is crucial for improving structural response to phenomena such as earthquakes. Ductility can be quantified in various ways: one approach is through the plasticity index, which relates plastic deformation to elastic deformation (μd = Δu/Δy), and another is through energy absorption capacity, calculated as the ratio between the area under the load–displacement curve up to ultimate deformation and the area under the curve up to plastic deformation (μE = Eu/Ey) (Figure 23). The corresponding values are provided in Table 9.

The corresponding values are reported in Table 9. Identifying the point at which damage prevents an element from being regarded as elastic is not straightforward. In the present tests, the elastic displacements (Δy) were similar for all concretes. At maximum load (Δu), the RV6 beam exhibited the highest plasticity index (μd = Δu/Δy), with a value of 2.81, which is 31.45% higher than that of the reference beam (C). In contrast, the R1 and R6M3 beams showed values 32.5% and 39.1% lower than those of beam C, respectively. These results indicate that increases in load-bearing capacity may be accompanied by reduced ductility; in this case, the RV6 beam’s higher tensile strength and enhanced bond capacity likely contributed to its superior overall performance.

Extending the analysis to the complete load–displacement curve beyond peak load, the area enclosed under the curve was 45.89% higher for the RV6 beam than for the reference concrete, and 23.396% higher for the R1 beam. Considering the energy accumulated in the elastic region relative to that accumulated in the plastic region, the RV6 beam again exhibited a value 44.5% higher than that of beam C. This ratio was similar for the remaining beams, with differences below 12.47%.

#### 2.8.4. Failures Mechanism and Crack Behaviour

The beams studied exhibit a similar number of cracks. All beams failed as expected, with failure occurring in the compressed zone and the development of a stabilized cracking regime in the lower zone. Similar observations have been reported by other authors [32,47,48]. Although this observation is qualitative, it is noted that in beams made with Portland cement, the distance between cracks is more consistent, and the stabilized cracking regime is reached earlier compared to beams made with alkali-activated concretes.

To complement the qualitative observations, this study carried out a quantitative crack assessment in the constant-moment region between the two loading points (L = 600 mm). The number of visible dominant flexural cracks in the tensile zone (n) can be observed in the post-test photographs (Figure 23), and the average crack spacing was estimated as s_avg_ = L/(n − 1). Table 13 reports the resulting metrics.

Based on the constant-moment region assessment (Table 12), the alkali-activated beams exhibited a denser flexural crack pattern (higher n and lower s_avg_) than the reference OPC beam, with RV6 showing the highest crack number and the smallest average spacing.

Figure 23 and Table 12 indicate that the reference beam C1 developed fewer dominant flexural cracks in the constant-moment region, with larger apparent crack openings, whereas beams R1, RV6 and R6M3 exhibited a more distributed cracking pattern (higher crack number and smaller average spacing) for the same reinforcement arrangement. A more distributed cracking pattern is commonly associated with reduced crack localization prior to ultimate compression crushing.

## 3. Conclusions

The following conclusions can be drawn from the results of this experimental work:

1. Feasibility and sensitivity to precursor/activator design. Residual diatomaceous earth can be effectively used as a silica-rich component in slag-based alkali-activated systems, achieving mechanical performance comparable to OPC concrete; however, strength development and overall performance remain strongly dependent on precursor characteristics (source, fineness/surface area and Si-Al chemistry) and activator dosage, confirming the need for mixture-specific optimization.
2. Mechanical properties at material scale. For the strength range investigated, the tensile and flexural strengths of the alkali-activated concrete were comparable to those expected for conventional concretes at similar compressive strength levels, and the measured elastic modulus remained within typical design-based estimates (EC2) and within the variability observed among mixtures.
3. Bond as a governing parameter for structural performance. Steel–concrete bond response varied markedly among mixtures and cannot be inferred from compressive strength alone. The results across slip levels and the observed failure modes highlight that mixture-dependent matrix characteristics affect interfacial shear transfer; therefore, bond performance should be explicitly considered when assessing reinforced alkali-activated concretes.
4. Member-level response and cracking. Reinforced beams manufactured with alkali-activated mixtures showed adequate flexural capacity for structural applications. Among the tested members, RV6 exhibited the most favorable overall response (higher moment capacity and deformation/energy absorption). Cracking was objectively compared in the constant-moment region using crack number and average spacing (Table 12), providing a serviceability-relevant metric; since crack widths were not instrumentally measured, crack opening statements are limited to qualitative interpretation from post-test observations.
5. Limitations and future work. This study prioritizes combined material-and member-scale validation. Further work is recommended to directly verify reaction and interfacial mechanisms through quantitative amorphous-content analysis and SEM/EDS of the binder matrix and bar–paste interface, and evaluate long-term durability and performance under controlled environmental exposure conditions.

Overall, slag-based alkali-activated concretes incorporating natural and residual diatomaceous earth achieved mechanical and structural performance comparable to OPC concrete when precursor characteristics and activator dosage were properly optimized. Bond response and cracking distribution in the constant-moment region showed clear mixture dependency and should be explicitly considered for reinforced applications, while RV6 provided the most favorable member-level response among the tested beams.

## Figures and Tables

**Table 1:** Chemical composition of raw materials (XRF, oxides in wt.%) and loss on ignition, PF (wt.%). Composition.

Elements	R1 (%)	R6 (%)	R7 (%)	R2M3 (%)	R6M3 (%)	RV6 (%)	RV7 (%)
PF	40.3480	42.4645	41.1770	42.8960	42.4160	39.5170	41.9610
O	19.7649	18.0403	18.7728	19.1399	19.5101	19.3169	19.0773
Na	1.1800	0.6650	0.7330	0.9570	1.1600	0.7510	1.0200
Mg	3.0110	2.6840	2.0710	5.3050	4.6380	1.9740	3.7370
Al	1.2500	0.5150	0.8980	0.9010	1.0300	0.8860	0.8150
Si	3.2680	1.7300	2.4000	2.8300	3.5010	2.6210	2.8210
P	0.0110	0.0086	0.0097	0.0500	0.0479	0.0130	0.0120
S	0.0958	0.0545	0.0662	0.0520	0.0554	0.0653	0.0758
Cl	0.0140	0.0100	0.0130	0.0130	0.0140	0.0110	0.0150
K	0.2250	0.0897	0.2240	0.1400	0.1590	0.2550	0.1200
Ca	29.9600	33.3900	32.8600	27.1900	26.8600	33.6800	29.8200
Ti	0.1500	0.0684	0.1010	0.1070	0.1210	0.1040	0.1090
V	0.0051	n. d.	0.0038	n. d.	n. d.	n. d.	n. d.
Cr	n. d.	n. d.	n. d.	0.0032	0.0047	0.0046	0.0030
Mn	0.2720	0.1350	0.1720	0.1870	0.2160	0.1520	0.1870
Fe	0.3190	0.1120	0.3560	0.1700	0.1930	0.4680	0.1710
Cu	0.0025	0.0025	0.0027	0.0025	0.0029	0.0029	0.0034
Zn	0.0038	0.0035	0.0030	0.0027	0.0032	0.0034	0.0035
Sr	0.0738	0.0239	0.1042	0.0259	0.0306	0.1357	0.0226
Y	0.0010	n. d.	n. d.	n. d.	0.0011	n. d.	n. d.
Zr	0.0071	0.0031	0.0066	0.0039	0.0041	0.0072	0.0034
Ba	0.0380	n. d.	0.0260	0.0240	0.0320	0.0320	0.0230

Notes: PF (Loss on Ignition): Obtained through thermogravimetric analysis. O: Stoichiometric oxygen of elements in the Na to Ba range. n. d.: Not detected.

**Table 2:** Leachate Results (5 g of non-dried solid in 50 mL of water I, according to standard DIN-38414-S4).

Sample	EC (mS/cm)	pH
R1	1.85	11.55
R6	1.10	10.79
R7	1.06	11.53
RV6	1.43	11.48
RV7	1.15	11.15
R2M3	1.11	10.76
R6M3	1.23	10.84

**Table 3:** Relative density and Blaine fineness of the natural diatoms studied.

Slag	Relative Density (g/cm^3^)	Blaine Fineness (cm^2^/g)
Diatomite 2	2.238	9558
Diatomite 3	2.262	10,601

**Table 4:** BET Surface Area and Pore Volume of the Materials Used.

Material	BET Surface Area (m^2^/g)	Pore Volume (cm^3^/g)
CEM I 52.5R	0.829	3.048 × 10^−3^
Diatomite M2	8.400	1.611 × 10^−2^
Diatomite M3	9.969	1.586 × 10^−2^
Diatomite V6	0.342	1.896 × 10^−2^
Slag	1.42	

**Table 5:** Oxide composition of precursors and binders determined by XRF (wt.%).

Diatom M2	Concentration	Diatom M3	Concentration	Wine Diatom	Concentration
H_2_O	4.33	H_2_O	4.361		
CO_2_	22.42	CO_2_	20.21	H_2_O	3.537
Na_2_O	0.075	Na_2_O	0.094	CO_2_	0.06791
MgO	0.401	MgO	0.413	Na_2_O	1
Al_2_O_3_	0.802	Al_2_O_3_	0.915	MgO	0.259
SiO_2_	46.03	SiO_2_	48.15	Al_2_O_3_	3.49
P_2_O_5_	0.036	P_2_O_5_	0.043	SiO_2_	84.42
SO_3_	0.295	SO_3_	0.2	P_2_O_5_	0.571
Cl	0.016	Cl	0.021	SO_3_	0.073
K_2_O	0.195	K_2_O	0.199	K_2_O	0.633
CaO	24.53	CaO	24.5	CaO	0.89
TiO_2_	0.0486	TiO_2_	0.0522	TiO_2_	0.801
MnO	0.0051	MnO	0.0058	V_2_O_5_	0.031
Fe_2_O_3_	0.412	Fe_2_O_3_	0.456	Cr_2_O_3_	0.015
CuO	0.0034	CuO	0.0044	MnO	0.0587
ZnO	0.0023	ZnO	0.0018	Fe_2_O_3_	4.046
SrO	0.3569	SrO	0.3382	CoO	0.0015
ZrO_2_	0.01588	ZrO_2_	0.0148	NiO	0.0059
				CuO	0.0071
				ZnO	0.00709
				Rb_2_O	0.0023
				SrO	0.0199
				Y_2_O_3_	0.0012
				ZrO_2_	0.0166
				Nb_2_O_5_	0.00526
				BaO	0.023

**Table 6:** Quantities used for the manufacture of alkaline activated mortars with NaOH at different concentrations.

Component	Quantity (g)			
Diatom	675	675	675	675
Sand	1350	1350	1350	1350
% Na_2_O	4% Na_2_O	6% Na_2_O	8% Na_2_O	10% Na_2_O
NaOH	34.8	52.3	69.7	87.1
Water	437.7	420.2	402.8	385.4

**Table 7:** NaOH molarity * corresponding to Na_2_O dosages used in Table 6 (based on NaOH and water masses).

Na_2_O Dosage	NaOH (g)	Water (g)	NaOH Molarity (M)
4% Na_2_O	34.8	437.7	2.0
6% Na_2_O	52.3	420.2	3.1
8% Na_2_O	69.7	402.8	4.3
10% Na_2_O	87.1	385.4	5.6

* Molarity computed as M = (m(NaOH)/40)/V, with V approximated from water mass assuming 1 kg ≈ 1 L for these dilute solutions.

**Table 8:** Mix proportions of alkali-activated and OPC concrete by weight (kg/m^3^).

Concrete	w/b	C (kg/m^3^)	GGBFS (kg/m^3^)	M2 (kg/m^3^)	M3 (kg/m^3^)	V (kg/m^3^)	Water (kg)	Sand (kg/m^3^)	Gravel (kg/m^3^)	NaOH (Kg)	w/NaOH
C	0.5	300	0	0	0	0	180	1100	900	0	0
R1	0.5	0	300	0	0	0	180	1100	900	26.90	6.69
R6	0.5	0	225	45	0	0	180	1100	900	26.90	6.69
R7	0.5	0	270	30	0	0	180	1100	900	26.90	6.69
RV6	0.5	0	225	0	0	45	180	1100	900	26.90	6.69
RV7	0.5	0	270	0	0	30	180	1100	900	26.90	6.69
R6 M3	0.5	0	225	0	45	0	180	1100	900	26.90	6.69
R7 M3	0.5	0	270	0	30	0	180	1100	900	26.90	6.69

C: Cement GGBFS: Ground Granulated Blast Furnace Slag; w: water; b: binder; M2: Natural diatom; M3: Natural diatom; V: Recycled diatom dried of wine.

**Table 9:** Pull-out test results in 150 mm cube specimens (28 days).

Mix	Bond Stress at Slips of 0.01, 0.1 and 1 mm			Meaning Bond Stress			Ultimate Bond Stress			Bar Stress (MPa)	Mode of Failure
	τ_b,0.01_ (MPa)	τ_b,0.1_ (MPa)	τ_b,1_ (MPa)	τ_bm_ (MPa)	τ_bm_/(f_c_)^0.5^	COV (%)	τ_bu_ (MPa)	τ_bu_/(f_c_)^0.5^	COV (%)		
C	6.18	12.38	20.33	12.96	1.90	5.30	21.72	3.19	3.74	435.4	Pull-out
R1	10.44	11.1	17.6	13.05	2.11	7.76	27.7	4.49	2.62	541.1	Pull-out
R6	15.83	16.25	19.61	17.23	2.89	1.56	20.73	3.48	2.36	415.0	Splitting
R7	8.36	9.02	14.46	10.61	1.87	3.45	20.06	3.53	0.59	321.1	Pull-out
RV6	13.13	13.42	14.44	13.66	2.18	4.65	16.22	2.59	3.1	324.8	Pull-out
R6M3	4.96	5.63	12.68	7.76	1.25	4.04	19.25	3.09	3.74	385.3	Pull-out

$\tau_{b,s}$ denotes bond stress at slip s (mm); $\tau_{bm}$ and $\tau_{bu}$ are the mean and ultimate bond stresses, respectively; normalized values are computed as τ/√f_c using fc in MPa.

**Table 10:** Dosages used in flexure tests.

Sample	a/f w/f	% Diatom Natural or Recycled	Cement	Blast Furnace Slag (g)	Diatom Recycled Wine Dried (g)	Diatom Natural M3 (g)	Water (g)	Sand (0/4)	Gravel	NaOH (g)	Water/ NaOH Waterglass/NaOH
C	0.55	0.00	13.86	0.00	0.00	0.00	7.62	27.72	41.58	0.00	0.00
R1	0.60	0.00	0.00	12.72	0.00	0.00	7.63	46.65	38.17	1.14	6.70
RV6	0.60	0.42	0.00	10.81	1.91	0.00	7.63	46.65	38.17	1.14	6.70
R6 M3	0.60	0.42	0.00	10.81	0.00	1.91	7.63	46.65	38.17	1.14	6.70

**Table 11:** Summary of tests results of load and final displacement.

Specimen Name	fcm (MPa)	fctm (MPa)	F (KN)	COV (%)	M_u_ (mKN)	Mteo (mKN)	d_max_ (mm)
C	37.44	5.05	64.88	2.3	19.46	17.39	39.59
R1	26.60	4.66	57.89	5.7	17.37	17.01	53.80
RV6	46.47	5.59	74.43	6.0	22.33	17.66	51.96
R6 M3	39.31	4.39	66.94	8.6	20.08	17.25	45.00

fcm: compressive strength. fctm: flexural tensile strength. F: Load apply by the press. COV: coefficient of variations. M_u_: Ultimate moment. Mteo: Theoretical calculated ultimate moment. d_max_: Mid-span deflection.

**Table 12:** Summary of tests results of deflection and energy under de curve load-deflection.

Specimen Name	D_y_	D_u_	μ_d_ = D_u_/D_y_	E_y_	E_u_	μ_E_ = E_u_/E_y_
	(mm)	(mm)		(KNmm)	(KNmm)	
C	10.21	21.80	2.14	313.9	1810.97	5.77
R1	11.44	16.49	1.44	369.2	2252.8	6.10
RV6	9.78	27.45	2.81	331.8	2768.2	8.34
R6M3	11.54	15.00	1.30	368.7	1863.5	5.05

**Table 13:** Crack metrics in the constant-moment region (L = 600 mm).

Beam	No. of Flexural Cracks, n	Average Crack Spacing, s_avg_ (mm)
C (C1)	6	120.0
R1	7	100.0
RV6	9	75.0
R6M3	6	120.0

## Data Availability

The original contributions presented in this study are included in the article. Further inquiries can be directed to the corresponding author.
